# Monocyte Count on Admission Is Predictive of Shunt-Dependent Hydrocephalus After Aneurysmal Subarachnoid Hemorrhage

**DOI:** 10.3389/fsurg.2022.879050

**Published:** 2022-04-28

**Authors:** Joshua A. Cuoco, Evin L. Guilliams, Brendan J. Klein, Mark R. Witcher, Eric A. Marvin, Biraj M. Patel, John J. Entwistle

**Affiliations:** ^1^Virginia Tech Carilion School of Medicine, Roanoke, VA, United States; ^2^Section of Neurosurgery, Carilion Clinic, Roanoke, VA, United States; ^3^School of Neuroscience, Virginia Polytechnic Institute and State University, Blacksburg, VA, United States; ^4^Neurointerventional Surgery, Department of Radiology, Carilion Clinic, Roanoke, VA, United States

**Keywords:** aneurysmal subarachnoid hemorrhage, immune system, hydrocephalus, monocyte, shunt-dependence

## Abstract

The authors sought to evaluate whether immunologic counts on admission were associated with shunt-dependent hydrocephalus following aneurysmal subarachnoid hemorrhage. A retrospective analysis of 143 consecutive patients with aneurysmal subarachnoid hemorrhage over a 9-year period was performed. A stepwise algorithm was followed for external ventricular drain weaning and determining the necessity of shunt placement. Data were compared between patients with and without shunt-dependent hydrocephalus. Overall, 11.19% of the cohort developed shunt-dependent hydrocephalus. On multivariate logistic regression analysis, acute hydrocephalus (OR: 61.027, 95% CI: 3.890–957.327; *p* = 0.003) and monocyte count on admission (OR: 3.362, 95% CI: 1.024–11.037; *p* = 0.046) were found to be independent predictors for shunt dependence. Receiver operating characteristic curve analysis for the prediction of shunt-dependent hydrocephalus confirmed that monocyte count exhibited an acceptable area under the curve (AUC = 0.737, 95% CI: 0.601–0.872; *p* < 0.001). The best predictive cutoff value to discriminate between successful external ventricular drain weaning and shunt-dependent hydrocephalus was identified as a monocyte count ≥0.80 × 10^3^/uL at initial presentation. These preliminary data demonstrate that a monocyte count ≥0.80 × 10^3^/uL at admission predicts shunt-dependent hydrocephalus in patients with aneurysmal subarachnoid hemorrhage; however, further large-scale prospective trials and validation are necessary to confirm these findings.

## Introduction

Aneurysmal subarachnoid hemorrhage (aSAH) remains a life-threatening disease with an annual worldwide incidence of 7.9 cases per 100,000 individuals (1, 2). Indeed, aSAH is associated with a multitude of secondary neurologic sequelae, such as seizure, vasospasm, delayed cerebral ischemia, hydrocephalus, and, importantly, shunt-dependence. Recent research has begun to reveal the association between neuroinflammation and hydrocephalus after injury (i.e., hemorrhage or infection). Various molecular mediators [e.g., tumor necrosis factor-alpha (TNF-α) (3), transforming growth factor-beta 1 (4, 5), thrombin-induced transforming growth factor-beta (6), interleukin-6 (IL-6) (7), nuclear factor kappa B (8)] in the peripheral blood or cerebrospinal fluid have been correlated with a high likelihood of developing post-hemorrhagic or post-infectious hydrocephalus as well as the clinical severity of symptomatology. In the context of aSAH, there is growing evidence to suggest that an immune-mediated inflammatory response in the central nervous system is a key mechanism of early brain injury following rupture and associated clinical sequelae (9, 10). Data from pre-clinical and clinical studies have demonstrated the activation and proliferation of various leukocyte subsets (e.g., neutrophils, monocytes) following aSAH in murine models and humans (9–14). Previously, our group found that a neutrophil count ≥9.80 × 10^3^/uL on admission was predictive of acute symptomatic hydrocephalus after aneurysmal subarachnoid hemorrhage in an adjusted multivariate logistic regression model (14). Secondary analysis demonstrated that 81.25% of patients who developed shunt dependence exhibited a neutrophil count ≥9.80 × 10^3^/uL on admission (p = 0.003) (14). Here, we sought to further investigate this preliminary finding in an independent analysis to evaluate for potential associations between immunologic counts on admission and shunt-dependent hydrocephalus following aSAH.

## Methods

### Patient Selection

**Figure 1:**
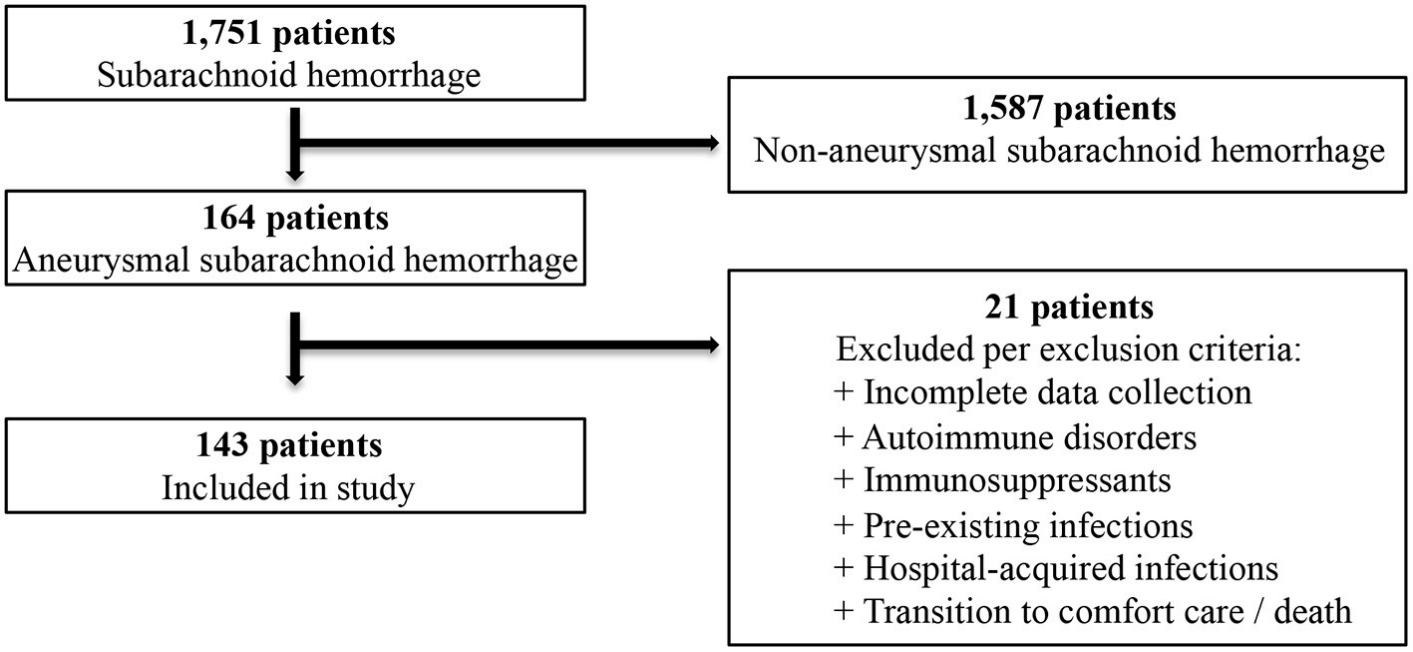
Flow chart depicting the process of patient selection.

The present study was conducted with the approval of the Carilion Clinic Institutional Review Board. Informed consent was waived due to the retrospective nature of the research. We performed a retrospective study and analysis of all consecutive patients with aSAH over a 9-year period (2012–2020) admitted to the neurosurgery service at Carilion Roanoke Memorial Hospital. Patients with cisternal subarachnoid hemorrhage diagnosed on computed tomography (CT) of the head with a confirmed cerebral aneurysm on CT angiography or digital subtraction angiography were eligible for inclusion. In order to avoid confounding, we excluded patients with documented infections prior to admission, those who developed in-hospital infectious complications as well as those with known autoimmune diseases or prescribed immunosuppressants. Additionally, we excluded patients who transitioned to palliative care or died during their hospitalization. Patients who met inclusion criteria found to have incomplete data upon chart review were also excluded (Figure 1).

### Management of Aneurysmal Subarachnoid Hemorrhage

Patients were managed according to the most recent *Guidelines for the Management of Aneurysmal Subarachnoid Hemorrhage*, as per the American Heart Association (15). Prompt treatment of the aneurysm was emphasized with either endovascular coil embolization or craniotomy for microsurgical clipping. Our multidisciplinary cerebrovascular team determined the appropriate treatment strategy for aneurysm obliteration on a case-by-case basis. Secondary sequelae of aSAH were managed aggressively in the intensive care unit including treatment of seizures, hydrocephalus, and vasospasm.

### Indications for External Ventricular Drain Placement

Both clinical and radiographic features were considered for the decision to place an external ventricular drain (EVD) at the time of admission, including clinical manifestations (e.g., altered mental status, lethargy, somnolence, or pupillary changes) and radiographic evidence indicative of acute hydrocephalus (e.g., ventriculomegaly, increase in temporal horn size, cisternal effacement, sulcal effacement). Lumbar drains were not utilized for cerebrospinal fluid diversion.

### Indications for Ventriculoperitoneal Shunt Placement

Our institution follows a stepwise algorithm to determine the necessity of shunt placement as described by Little et al. (16). External ventricular drains were initially set to 15 cm H_2_O after placement until the aneurysm was secured. Once secured, the EVD was opened between 0 and 10 cm H_2_O dependent upon clinical symptomatology attributable to hydrocephalus. Weaning of the EVD typically commenced within 1 week from the date of placement. For patients who developed symptomatic vasospasm requiring intervention, EVD weaning was postponed until vasospastic symptomatology had resolved. EVD weaning consisted of raising the height of the external collection reservoir over the course of several days by 5 cm H_2_O or less each day and to at least 25 cm H_2_O prior to clamping. The EVD was clamped for 24–48 h with continuous monitoring of intracranial pressure, frequent neurochecks, and a repeat CT scan to assess for recurrent hydrocephalus prior to removal. The EVD was reopened for the development of a new neurologic deficit or if intracranial pressure sustained above 22 mm Hg for > 10 min without stimulation. Weaning of the EVD was not attempted more than twice prior to considering shunt placement. Patients who tolerated weaning and removal of the EVD but subsequently developed recurrent clinical symptomatology consistent with hydrocephalus underwent a repeat CT scan and a lumbar puncture to evaluate for elevated pressure (> 22 cm H_2_O). Patients who failed EVD weaning, exhibited progressive enlargement of the ventricular system, or had elevated opening pressures via lumbar puncture were deemed candidates for placement of a ventriculoperitoneal shunt.

### Clinical and Laboratory Parameters

We analyzed data on demographic parameters (age, sex, ethnicity), clinical status (Hunt and Hess grade), radiographic imaging (modified Fisher score, presence of intraventricular hemorrhage, aneurysm morphology), and laboratory data (neutrophil, lymphocyte, monocyte, hemoglobin A1C) on admission. Treatment modality (endovascular coil embolization or open craniotomy for microsurgical clipping) and incidence and severity of vasospasm during hospitalization were assessed as well as patients requiring ventriculoperitoneal shunt placement prior to discharge. Laboratory data utilized for analysis was obtained immediately upon admission. Monocyte count was recorded from the first venous blood test on admission expressed as 10^3^ per microliter (10^3^/uL). Angiographic vasospasm was graded based upon digital subtraction angiography images and according to severity as follows: mild (0–33%), moderate (34–66%), or severe (67–100%) vessel narrowing of any vessel within the cerebrovasculature irrespective of the patient's corresponding neurologic deficit. The primary outcome of this study was to evaluate for potential associations between immunologic counts on admission and shunt-dependent hydrocephalus in aSAH patients.

### Statistical Analysis

XL STAT (Addinsoft) software was used to perform the statistical analyses. Chi-square test, Fisher's exact test, or Student's *t*-test were utilized to analyze clinical and laboratory data as appropriate. All variables with *p*-values < 0.05 in univariate analysis were included in the final multivariate logistic regression model. Vasospasm was dichotomized for multivariate analysis as none or mild vasospasm vs. moderate or severe vasospasm. We evaluated for interactions among all of the independent variables in the final model. Receiver operating characteristic (ROC) curve analysis was utilized to determine associations of monocyte count on admission with the primary outcome and evaluate the optimal cutoff value for outcome prediction. A *p*-value < 0.05 was considered statistically significant.

## Results

### Demographics and Clinical Features

Over the 9-year study period, 1,751 patients were diagnosed with subarachnoid hemorrhage of which 164 patients had aneurysmal subarachnoid hemorrhage. After applying inclusion and exclusion criteria, 143 patients met the eligibility criteria. The average documented time to presentation was 11.75 h. The majority of patients were female (81.82%) with a mean age of 54.99 ± 14.04 years. Of this cohort, Caucasians and African Americans represented 72.02% and 23.08% of patients, respectively. The vast majority of aneurysms (90.91%) were localized to the anterior circulation. Specifically, the anterior cerebral arteries (i.e., pre-communicating segment, communicating segment, post-communicating segment, pericallosal branch) represented the most common location of rupture (49.65%). Endovascular coil embolization was utilized in 81.12% of cases with the remaining undergoing open craniotomy for microsurgical clipping. A Hunt and Hess grade of two was most common representing 46.85% of patients while 57.34% exhibited a modified Fisher score of three. Intraventricular hemorrhage was observed in 39.86% of cases.

### Shunt-Dependent Hydrocephalus

Acute symptomatic hydrocephalus was diagnosed in 56 patients (39.16%): all of which required emergent EVD placement for cerebrospinal fluid diversion. Of this cohort, 28.57% of patients failed EVD weaning and requirement shunt placement. Overall, 16 patients (11.19%) developed shunt-dependent hydrocephalus. Univariate analysis indicated multiple factors associated with shunt dependence following aSAH, including: intraventricular hemorrhage [46/127 (36.22%) vs. 11/16 (68.75%); *p* = 0.012], acute hydrocephalus [40/127 (31.50%) vs. 16/16 (100%); *p* < 0.001], incidence of vasospasm [9/127 (7.08%) vs. 7/16 (43.75%); *p* < 0.001], neutrophil count on admission [9.69 vs. 13.03; *p* = 0.002], monocyte count on admission (0.67 vs. 1.21; *p* < 0.001), prior ischemic stroke [10/127 (7.87%) vs. 6/16 (37.50%); *p* < 0.001], and a history of diabetes mellitus [4/127 (3.15%) vs. 4/16 (25.00%); *p* = 0.006] (Table 1).

**Table 1:** Univariate analysis of predictors of shunt dependence after aneurysmal subarachnoid hemorrhage.

		**Shunt dependence**			
	**Yes**		**No**		
	**Mean / Count**	**% or SD**	**Mean / Count**	**% or SD**	***p*-value**
**Characteristics**					
**Age (yrs)**	52.13	13.50	55.35	14.12	0.194
**Sex**					0.305
Male	1	6.25%	25	19.69%	
Female	15	93.75%	102	80.31%	
**Ethnicity**					0.184
Caucasian	9	56.25%	94	74.02%	
African American	7	43.75%	26	20.47%	
Hispanic	0	0.00%	6	4.72%	
Asian	0	0.00%	1	0.79%	
**Aneurysm location**					0.362
Anterior circulation	16	100.00%	114	89.76%	
Posterior circulation	0	0.00%	13	10.24%	
**Aneurysm specific location**					0.389
ACA	7	43.75%	64	50.39%	
ICA / PCOM	6	37.50%	37	29.13%	
MCA	3	18.75%	13	10.24%	
PC	0	0.00%	13	10.24%	
**Aneurysm size (mm)**	6.68	3.52	5.59	3.33	0.111
**Aneurysm treatment**					0.18
Endovascular coiling	11	68.75%	105	82.68%	
Surgical clipping	5	31.25%	22	17.32%	
**Ventilation on admission**	4	25.00%	17	13.39%	0.256
**Hunt and Hess grade**					0.132
1	0	0.00%	12	9.45%	
2	4	25.00%	63	49.60%	
3	9	56.25%	38	29.92%	
4	2	12.50%	9	7.09%	
5	1	6.25%	5	3.94%	
**Modified Fisher grade**					0.057
0	0	0.00%	1	0.79%	
1	0	0.00%	3	2.36%	
2	1	6.25%	13	10.24%	
3	5	31.25%	77	60.63%	
4	10	62.50%	33	25.98%	
**Intraventricular hemorrhage**	11	68.75%	46	36.22%	0.012
^*^Acute hydrocephalus	16	100.00%	40	31.50%	<0.001
**Average mean flow velocity (cm/sec)**	134.70	15.60	130.11	33.10	0.293
**Severity of vasospasm**					<0.001
None	9	56.25%	118	92.92%	
Mild	0	0.00%	0	0.00%	
Moderate	3	18.75%	3	2.36%	
Severe	4	25.00%	6	4.72%	
**Laboratory values on admission**					
Neutrophil count (x 10^3^/uL)	13.03	3.31	9.69	4.52	0.002
Lymphocyte count (x 10^3^/uL)	2.15	1.66	1.87	1.30	0.213
Monocyte count (x 10^3^/uL)	1.21	1.09	0.67	0.48	<0.001
NLR	7.82	3.42	8.93	13.19	0.370
LMR	2.24	1.24	4.80	6.39	0.057
MNLR	8.43	3.67	9.57	14.45	0.378
Hemoglobin A1C (%)	5.75	0.61	5.58	0.52	0.199
**Past medical history**					
Body mass index (kg/m)	29.44	3.50	28.68	5.32	0.291
Hypertension	12	75.00%	61	48.03%	0.062
Hyperlipidemia	3	18.75%	19	14.96%	0.714
Ischemic stroke	6	37.50%	10	7.87%	<0.001
Diabetes mellitus	4	25.00%	4	3.15%	0.006
Peripheral vascular disease	1	6.25%	2	1.57%	0.301
Chronic kidney disease	2	12.50%	6	4.72%	0.22
Hyperthyroidism	1	6.25%	4	3.15%	0.452
Hypothyroidism	0	0.00%	3	2.36%	1.00

*SD, standard deviation; yrs, years; ACA, anterior cerebral artery; ICA, internal carotid artery; PCOM, posterior communicating artery; MCA, middle cerebral artery; PC, posterior circulation; NLR, neutrophil-to-lymphocyte ratio; LMR, lymphocyte-to-monocyte ratio; MNLR, monocyte-neutrophil-to-lymphocyte ratio*.

* *Acute hydrocephalus indicates symptomatic hydrocephalus requiring external ventricular drain placement on admission*.

Regression analysis demonstrated that monocyte count on admission was positively associated with shunt-dependent hydrocephalus (OR: 2.838, 95% CI: 1.242–6.483; *p* = 0.013). After adjusting the model for intraventricular hemorrhage, acute hydrocephalus, moderate to severe vasospasm, neutrophil count on admission, prior ischemic stroke, and a history of diabetes mellitus, monocyte count on admission remained an independent predictor for shunt-dependent hydrocephalus following aSAH [OR: 3.362, 95% CI: 1.024–11.037; *p* = 0.046). Moreover, acute hydrocephalus (OR: 61.027, 95% CI: 3.890–957.327; *p* = 0.003) was independently associated with shunt dependence. All other variables failed to retain statistical significance in the multivariate model (Table 2).

**Table 2:** Multivariate logistic regression analysis with adjusted odds ratios of shunt dependence after aneurysmal subarachnoid hemorrhage.

**Variable**	**Adjusted odds ratio**	**95% Confidence interval**	***p*-value**
Intraventricular hemorrhage	0.638	0.152–2.678	0.539
^*^Acute hydrocephalus	61.027	3.890–957.327	0.003
Moderate to severe vasospasm	4.954	0.988–24.825	0.052
Neutrophil count	0.993	0.845–1.168	0.936
Monocyte count	3.362	1.024–11.037	0.046
History of ischemic stroke	0.134	0.006–2.938	0.202
History of diabetes mellitus	0.169	0.002–12.306	0.417

* *Acute hydrocephalus indicates symptomatic hydrocephalus requiring external ventricular drain placement on admission*.

**Figure 2:**
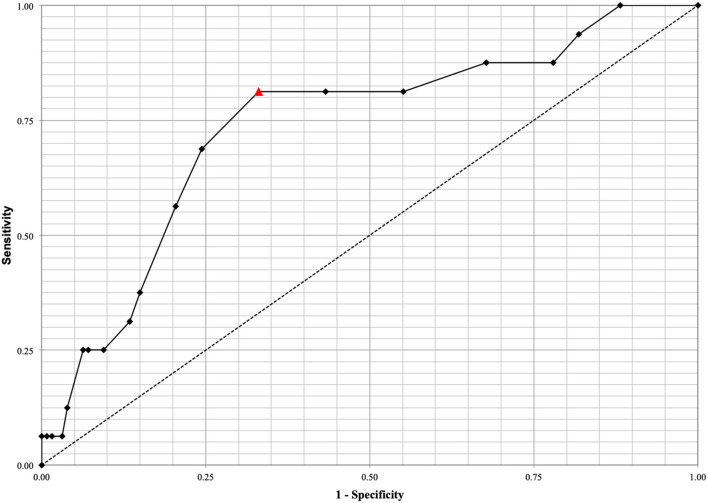
ROC analysis for the prediction of shunt-dependent hydrocephalus demonstrated that monocyte count exhibits an acceptable area under the curve (AUC = 0.737, 95% CI: 0.601–0.872, p < 0.001). The best predictive cutoff value to discriminate between successful EVD weaning and shunt-dependent hydrocephalus was identified at a monocyte count ≥0.80 × 10^3^/uL with a sensitivity and specificity of 81.25% and 66.93%, respectively. The optimal cutoff value of monocyte count on admission is depicted by the red triangle.

ROC analysis for the prediction of shunt-dependent hydrocephalus demonstrated that monocyte count exhibited an acceptable area under the curve (AUC = 0.737, 95% CI: 0.601–0.872; *p* < 0.001) (Figure 2). The best predictive cutoff value to discriminate between successful EVD weaning and shunt-dependent hydrocephalus was identified as a monocyte count ≥0.80 × 10^3^/uL at initial presentation with a sensitivity and specificity of 81.25and 66.93%, respectively.

Two supplemental multivariate analyses were performed on the cohort (*n* = 143) and included all statistically significant variables identified in the original univariate analysis with the exclusion of the acute hydrocephalus variable in the first additional analysis and the intraventricular hemorrhage variable in the second additional analysis. This was performed to investigate the statistical significance of the other variables given the fact that acute hydrocephalus was highly associated with shunt-dependence in the original multivariate analysis. In the first multivariate analysis (acute hydrocephalus variable excluded), moderate to severe vasospasm was significantly associated with shunt-dependence (OR: 6.397, 95% CI: 1.532–26.703; *p* = 0.011) (Table 3). Monocyte count trended toward significance (OR: 3.659, 95% CI: 0.93–14.4; *p* = 0.064). Best model selection based upon likelihood ratio associated moderate to severe vasospasm (OR: 6.794, 95% CI: 1.927–23.946; *p* = 0.003) and monocyte count (OR: 4.224, 95% CI: 1.164–15.33; *p* = 0.028) with shunt-dependence. In the second multivariate analysis (intraventricular hemorrhage variable excluded), acute hydrocephalus was significantly associated with shunt-dependence (OR: 45.868, 95% CI: 3.461–607.93; *p* = 0.004) (Table 4). Monocyte count trended toward significance (OR: 5.113, 95% CI: 0.978–26.732; *p* = 0.053). Best model selection based upon likelihood ratio associated acute hydrocephalus (OR: 53.9, 95% CI: 3.179–913.849; *p* = 0.006) and moderate to severe vasospasm (OR: 5.114, 95% CI: 1.352–19.35; *p* = 0.016) with shunt-dependence.

**Table 3:** Multivariate logistic regression analysis with adjusted odds ratios of shunt dependence after aneurysmal subarachnoid hemorrhage (acute hydrocephalus variable excluded).

**Variable**	**Adjusted Odds Ratio**	**95% Confidence Interval**	***p*-value**
Intraventricular hemorrhage	1.696	0.488–5.896	0.406
Moderate to severe vasospasm	6.397	1.532–26.703	0.011
Neutrophil count	1.054	0.931–1.194	0.407
Monocyte count	3.659	0.93–14.4	0.064
History of ischemic stroke	0.125	0.005–3.039	0.201
History of diabetes mellitus	0.369	0.012-11.405	0.569

**Table 4:** Multivariate logistic regression analysis with adjusted odds ratios of shunt dependence after aneurysmal subarachnoid hemorrhage (intraventricular hemorrhage variable excluded).

**Variable**	**Adjusted odds ratio**	**95% Confidence interval**	***p*-value**
^*^Acute hydrocephalus	45.868	3.461–607.93	0.004
Moderate to severe vasospasm	4.13	0.868–19.663	0.075
Neutrophil count	0.993	0.843–1.169	0.93
Monocyte count	5.113	0.978–26.732	0.053
History of ischemic stroke	0.107	0.005–2.385	0.158
History of diabetes mellitus	0.264	0.005–14.365	0.513

* *Acute hydrocephalus indicates symptomatic hydrocephalus requiring external ventricular drain placement on admission*.

Additional sub-cohort statistical analyses were performed for all patients exhibiting acute hydrocephalus (*n* = 56) as well as intraventricular hemorrhage (*n* = 57). Univariate analysis of the acute hydrocephalus sub-cohort indicated sex (*p* = 0.043), incidence of vasospasm (*p* = 0.026), monocyte count on admission (*p* = 0.013), and history of diabetes mellitus (*p* = 0.02) to be associated with shunt-dependence (Table 5). Univariate analysis of the intraventricular hemorrhage sub-cohort indicated acute hydrocephalus (*p* = 0.005), incidence of vasospasm (*p* = 0.007), neutrophil count on admission (*p* = 0.018), monocyte count on admission (*p* = 0.003), and prior ischemic stroke (*p* = 0.027) to be associated with shunt-dependence (Table 6). Multivariate analyses failed to reveal statistically significant relationships given the small sample size of each sub-cohort.

**Table 5:** Sub-cohort univariate analysis of predictors of shunt dependence after aneurysmal subarachnoid hemorrhage in patients with acute hydrocephalus.

		**Shunt dependence**			
	**Yes**		**No**		
	**Mean / Count**	**% or SD**	**Mean / Count**	**% or SD**	***p*-value**
**Characteristics**					
**Age (yrs)**	52.13	13.50	52.95	16.46	0.43
**Sex**					0.043
Male	1	6.25%	14	35.00%	
Female	15	93.75%	26	65.00%	
**Ethnicity**					0.343
Caucasian	9	56.25%	28	70.00%	
African American	7	43.75%	9	22.50%	
Hispanic	0	0.00%	2	5.00%	
Asian	0	0.00%	1	2.50%	
**Aneurysm location**					0.315
Anterior circulation	16	100.00%	36	90.00%	
Posterior circulation	0	0.00%	4	10.00%	
**Aneurysm specific location**					0.55
ACA	7	43.75%	19	47.50%	
ICA / PCOM	6	37.50%	11	27.50%	
MCA	3	18.75%	6	15.00%	
PC	0	0.00%	4	10.00%	
**Aneurysm size (mm)**	6.68	3.52	5.89	4.00	0.248
**Aneurysm treatment**					0.756
Endovascular coiling	11	68.75%	29	72.50%	
Surgical clipping	5	31.25%	11	27.50%	
**Ventilation on admission**	4	25.00%	13	32.50%	0.751
**Hunt and Hess grade**					0.678
1	0	0.00%	1	2.50%	
2	4	25.00%	5	12.50%	
3	9	56.25%	21	52.50%	
4	2	12.50%	8	20.00%	
5	1	6.25%	5	12.50%	
**Modified Fisher grade**					0.955
0	0	0.00%	0	0.00%	
1	0	0.00%	0	0.00%	
2	1	6.25%	2	5.00%	
3	5	31.25%	14	35.00%	
4	10	62.50%	24	60.00%	
**Intraventricular hemorrhage**	11	68.75%	26	65.00%	1
**Average mean flow velocity (cm/sec)**	134.70	15.60	130.94	37.09	0.349
**Severity of vasospasm**					0.026
None	9	56.25%	35	87.50%	
Mild	0	0.00%	0	0.00%	
Moderate	3	18.75%	1	2.50%	
Severe	4	25.00%	4	10.00%	
**Laboratory values on admission**					
Neutrophil count (x 10^3^/uL)	13.03	3.31	12.25	3.91	0.243
Lymphocyte count (x 10^3^/uL)	2.15	1.66	1.68	1.40	0.143
Monocyte count (x 10^3^/uL)	1.21	1.09	0.75	0.43	0.013
NLR	7.82	3.42	13.45	20.58	0.142
LMR	2.24	1.24	3.96	6.42	0.147
MNLR	8.43	3.67	14.47	22.94	0.151
Hemoglobin A1C (%)	5.75	0.61	5.68	0.55	0.392
**Past medical history**					
Body mass index (kg/m)	29.44	3.50	28.75	5.61	0.325
Hypertension	12	75.00%	25	62.50%	0.534
Hyperlipidemia	3	18.75%	6	15.00%	0.705
Ischemic stroke	6	37.50%	7	17.50%	0.109
Diabetes mellitus	4	25.00%	1	2.50%	0.02
Peripheral vascular disease	1	6.25%	1	2.50%	0.494
Chronic kidney disease	2	12.50%	3	7.50%	0.617
Hyperthyroidism	1	6.25%	2	5.00%	1
Hypothyroidism	0	0.00%	2	5.00%	1

*SD, standard deviation; yrs, years; ACA, anterior cerebral artery; ICA, internal carotid artery; PCOM, posterior communicating artery; MCA, middle cerebral artery; PC, posterior circulation; NLR, neutrophil-to-lymphocyte ratio; LMR, lymphocyte-to-monocyte ratio; MNLR, monocyte-neutrophil-to-lymphocyte ratio*.

**Table 6:** Sub-cohort univariate analysis of predictors of shunt dependence after aneurysmal subarachnoid hemorrhage in patients with intraventricular hemorrhage.

		**Shunt dependence**			
	**Yes**		**No**		
	**Mean / Count**	**% or SD**	**Mean / Count**	**% or SD**	***p*-value**
**Characteristics**					
**Age (yrs)**	52.64	11.25	54.63	15.02	0.341
**Sex**					0.261
Male	1	9.09%	13	28.26%	
Female	10	90.91%	33	71.74%	
**Ethnicity**					0.595
Caucasian	6	54.55%	32	69.57%	
African American	5	45.45%	12	26.09%	
Hispanic	0	0.00%	1	2.17%	
Asian	0	0.00%	1	2.17%	
**Aneurysm location**					0.585
Anterior circulation	11	100.00%	40	86.96%	
Posterior circulation	0	0.00%	6	13.04%	
**Aneurysm specific location**					0.589
ACA	5	45.46%	19	41.31%	
ICA / PCOM	3	27.27%	13	28.26%	
MCA	3	27.27%	8	17.39%	
PC	0	0.00%	6	13.04%	
**Aneurysm size (mm)**	7.73	3.53	6.01	4.21	0.108
**Aneurysm treatment**					0.455
Endovascular coiling	7	63.64%	35	76.09%	
Surgical clipping	4	36.36%	11	23.91%	
**Ventilation on admission**	4	36.36%	14	30.43%	0.728
**Hunt and Hess grade**					0.78
1	0	0.00%	6	13.04%	
2	1	9.09%	4	8.70%	
3	7	63.64%	25	54.35%	
4	2	18.18%	6	13.04%	
5	1	9.09%	5	10.87%	
**Modified Fisher grade**					0.261
0	0	0.00%	0	0.00%	
1	0	0.00%	0	0.00%	
2	1	9.09%	13	28.26%	
3	0	0.00%	0	0.00%	
4	10	90.91%	33	71.74%	
^*^Acute hydrocephalus	11	100.00%	26	56.52%	0.005
**Average mean flow velocity (cm/sec)**	134.73	16.07	125.91	35.33	0.213
**Severity of vasospasm**					0.007
None	5	45.46%	40	86.96%	
Mild	0	0.00%	0	0.00%	
Moderate	3	27.27%	1	2.17%	
Severe	3	27.27%	5	10.87%	
**Laboratory values on admission**					
Neutrophil count (x 10^3^/uL)	14.02	2.80	10.68	4.97	0.018
Lymphocyte count (x 10^3^/uL)	2.15	1.97	1.86	1.42	0.284
Monocyte count (x 10^3^/uL)	1.36	1.26	0.72	0.43	0.003
NLR	8.83	3.36	9.46	9.17	0.412
LMR	1.91	1.15	4.38	5.93	0.089
MNLR	9.55	3.65	10.07	9.71	0.43
Hemoglobin A1C (%)	5.67	0.70	5.71	0.52	0.435
**Past medical history**					
Body mass index (kg/m)	29.27	3.23	29.25	5.78	0.495
Hypertension	10	90.91%	28	60.87%	0.079
Hyperlipidemia	2	18.18%	10	21.74%	1
Ischemic stroke	5	45.45%	6	13.04%	0.027
Diabetes mellitus	2	18.18%	3	6.52%	0.244
Peripheral vascular disease	1	9.09%	2	4.35%	0.481
Chronic kidney disease	2	18.18%	3	6.52%	0.244
Hyperthyroidism	0	0.00%	2	4.35%	1
Hypothyroidism	0	0.00%	1	2.17%	1

*SD, standard deviation; yrs, years; ACA, anterior cerebral artery; ICA, internal carotid artery; PCOM, posterior communicating artery; MCA, middle cerebral artery; PC, posterior circulation; NLR, neutrophil-to-lymphocyte ratio; LMR, lymphocyte-to-monocyte ratio; MNLR, monocyte-neutrophil-to-lymphocyte ratio*.

* *Acute hydrocephalus indicates symptomatic hydrocephalus requiring external ventricular drain placement on admission*.

## Discussion

In the present study, we identified independent predictors for shunt dependence in a consecutive cohort of patients with aSAH. On multivariate logistic regression analysis, monocyte count on admission and acute hydrocephalus were found to be independent risk factors for shunt dependence after aSAH. These data were irrespective of the clinical or radiographic grades of injury burden.

Thirty-nine percent of patients required placement of an EVD for treatment of acute symptomatic hydrocephalus. Of the cohort requiring cerebrospinal fluid diversion, 28.57% of patients developed shunt dependence. Overall, 11.19% included in the study developed shunt-dependent hydrocephalus. Our rate of shunt dependence is similar to prior reports, which range between 6 and 67% (17–19). Prior studies have found an association between shunt dependence and a multitude of clinical and radiographic variables including age (17), sex (18), Hunt and Hess grade ≥4 (19), Fisher grade 4 (20), bicaudate index of at least 0.20 (20), acute hydrocephalus (17–19), presence of intraventricular hemorrhage (19, 20), posterior circulation aneurysms (17, 19), giant aneurysms (17), vasospasm (18), ventilation on admission (17), sustained systemic inflammatory response syndrome criteria (18) ≥78 cc/day of cerebrospinal fluid drainage (21) as well as ≥1,500 cc of cerebrospinal fluid drainage during the first week after ictus (22), among others. Our univariate analysis corroborated several of these traditional factors associated with shunt dependence including acute hydrocephalus, presence of intraventricular hemorrhage, and incidence of vasospasm. However, after accounting for monocyte count on admission, only acute hydrocephalus retained statistical significance in the adjusted multivariate model. Additional novel findings of the present report are the associations of neutrophil count on admission, prior ischemic stroke, and a history of diabetes mellitus with shunt dependence; however, all of these variables failed to retain statistical significance in the multivariate model.

These preliminary data invoke several questions regarding the association between monocyte count and shunt dependence in aSAH patients including: (i) what are the clinical implications of increased monocyte count on admission, (ii) can monocyte count on admission be used as a biomarker for shunt-dependent hydrocephalus, and (iii) do monocytes contribute to the inflammatory changes associated with impaired cerebrospinal fluid dynamics following aSAH? Regarding clinical implications, our preliminary data have shown that monocyte count on admission predicts shunt dependence after aSAH. Moreover, prior studies have shown an association between monocyte counts on admission or monocyte-based inflammatory indices with unfavorable functional outcomes and cerebral infarction (23, 24). Feghali et al. (23) showed that the monocyte-neutrophil-to-lymphocyte ratio and lymphocyte-to-monocyte ratio, but not the neutrophil-to-lymphocyte ratio, to be independently associated with unfavorable functional outcomes (mRS of 3–6) at 12–18 months following discharge. Moreover, the authors reported the lymphocyte-to-monocyte ratio to be significantly associated with vasospasm (23). Unda et al. (24) found a relationship between monocyte count on admission with cerebral infarction and unfavorable functional outcomes (mRS of 3–6) at 12 months after discharge. Indeed, the present report along with the aforementioned studies has begun to demonstrate a preliminary association between monocytes and clinical sequelae following aSAH.

Immune characterization in aSAH has found that monocytes exhibit a fundamental role in propagating a pro-inflammatory state after aneurysm rupture (10–12). Data from pre-clinical and clinical studies have demonstrated the activation and proliferation of pro-inflammatory monocytes following aSAH in murine models and humans (10–12). Gris et al. (10) found an early increase in recruitment of pro-inflammatory monocytes within the first 48 h after aSAH in a murine model. Moreover, in a murine model, the authors found an increase of circulating and brain monocytes and brain neutrophils following aSAH (10). Mohme et al. (11) showed that systemic and intrathecal immune activation and secondary immune dissemination of non-classical monocytes (i.e., pro-inflammatory, CD14^+^, CD16^++^) exhibit a critical role in aSAH-induced neuroinflammation and delayed cerebral ischemia in humans. Moreover, in the same study, *ex-vivo* analysis demonstrated that non-classical monocytes were the major monocytic cell population in the early phase (day 1–4) after aSAH (11). Korostynski et al. (12) found that rupture of an intracranial aneurysm significantly increases monocyte activity while depressing the lymphocyte response in both cellular composition and gene expression profiles. These data have implicated monocytes in the pathogenesis of neuroinflammation following aSAH.

The etiopathogenesis of shunt dependence following aSAH is likely a multifactorial process (3–8, 18). In the context of neuroinflammation, it is known that non-classical monocytes are the main source of pro-inflammatory cytokines, such as TNF- α, IL-6, and interleukin 1 beta: all of which are elevated in serum and cerebrospinal fluid following aSAH and contribute to the inflammatory process (18, 25–28). Moreover, elevated cerebrospinal fluid IL-6 is a known independent predictor for shunt dependence after aSAH (7). Might non-classical monocytes be responsible, to some degree, for neuroinflammation and consequential shunt dependence following aSAH? Future prospective studies examining non-classical monocyte levels within the cerebrospinal fluid and the expression of pro-inflammatory cytokines in the context of aSAH may determine the validity of this relationship.

There are several limitations to our study. This study lacks a prospective design and is limited to a single-center experience. Despite accounting for confounding variables, our findings may still overestimate or underestimate causal relationships. Although our study cohort had a reasonable number of subjects, an increased sample size may have yielded additional statistically significant associations. We limited our investigation to laboratory data on the date of admission. We cannot decipher whether only the acute increase in monocyte count on admission exhibits predictive value or if additional changes in monocyte count in the days or weeks following aneurysmal rupture plays any role. Moreover, we could not definitively determine time to presentation. Although the average documented time to presentation was 11.75 h, this was entirely dependent upon retrospective chart review including physician, resident, nurse, emergency medical technician, and transfer center notes. We could not determine if such information was obtained from the patient, family, or other means. As such, this is a limitation of the retrospective design of our study, which could be addressed with a prospective design in the future. Lastly, several studies in the literature have identified intraventricular hemorrhage as a risk factor for the development of shunt-dependent hydrocephalus (19, 20). While our univariate analysis confirmed this relationship, intraventricular hemorrhage failed to retain statistical significance in our multivariate analysis. We initially attributed this to the fact that intraventricular hemorrhage and acute hydrocephalus were entered together in the multivariate analysis with acute hydrocephalus becoming more statistically significant and intraventricular hemorrhage losing significance consequentially. However, a secondary multivariate analysis with the exclusion of the acute hydrocephalus variable failed to reestablish intraventricular hemorrhage as a statistically significant factor. An additional multivariate analysis with the exclusion of three variables (i.e., acute hydrocephalus, vasospasm, monocyte count on admission) reestablished intraventricular hemorrhage (*p* = 0.05) as a statistically significant predictor of shunt-dependence.

## Conclusions

In our retrospective study, a monocyte count ≥0.80 × 10^3^/uL at admission was found to be predictive of shunt-dependent hydrocephalus in patients with aSAH. Knowledge of monocyte count on admission may help predict the occurrence of shunt-dependent hydrocephalus; however, these data should be viewed as preliminary results as the validity of this association will be dependent on future larger prospective studies.

## Data Availability Statement

The datasets presented in this article are not readily available because these data are protected by the Institutional Review Board of Carilion Clinic (IRB-20-1059). Requests to access the datasets should be directed to JC, jacuoco@carilionclinic.org.

## Ethics Statement

The studies involving human participants were reviewed and approved by Institutional Review Board of Carilion Clinic (IRB 20-1059; Approved on 10/13/2020). Written informed consent for participation was not required for this study in accordance with the national legislation and the institutional requirements.

## Author Contributions

JC, EG, BK, MW, EM, BP, and JE: conceptualization, methodology, validation, formal analysis, investigation, resources, writing–review and editing, and visualization. JC and EG: data curation. JC: writing–original draft preparation. MW, EM, BP, and JE: supervision. JE: project administration. All authors contributed to the article and approved the submitted version.

## Conflict of Interest

The authors declare that the research was conducted in the absence of any commercial or financial relationships that could be construed as a potential conflict of interest.

## Publisher's Note

All claims expressed in this article are solely those of the authors and do not necessarily represent those of their affiliated organizations, or those of the publisher, the editors and the reviewers. Any product that may be evaluated in this article, or claim that may be made by its manufacturer, is not guaranteed or endorsed by the publisher.
